# Prediction of protein–ligand binding affinity via deep learning models

**DOI:** 10.1093/bib/bbae081

**Published:** 2024-03-05

**Authors:** Huiwen Wang

**Affiliations:** School of Physics and Engineering, Henan University of Science and Technology, Luoyang 471023, China

**Keywords:** protein–ligand binding affinity, accurate prediction, database, input representation, deep learning model

## Abstract

Accurately predicting the binding affinity between proteins and ligands is crucial in drug screening and optimization, but it is still a challenge in computer-aided drug design. The recent success of AlphaFold2 in predicting protein structures has brought new hope for deep learning (DL) models to accurately predict protein–ligand binding affinity. However, the current DL models still face limitations due to the low-quality database, inaccurate input representation and inappropriate model architecture. In this work, we review the computational methods, specifically DL-based models, used to predict protein–ligand binding affinity. We start with a brief introduction to protein–ligand binding affinity and the traditional computational methods used to calculate them. We then introduce the basic principles of DL models for predicting protein–ligand binding affinity. Next, we review the commonly used databases, input representations and DL models in this field. Finally, we discuss the potential challenges and future work in accurately predicting protein–ligand binding affinity via DL models.

## INTRODUCTION

**Figure 1:**
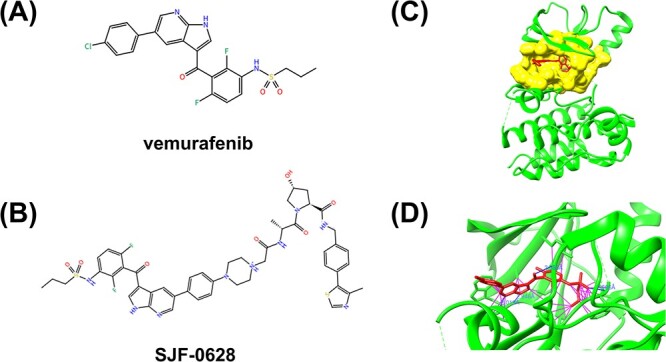
(**A**) The 2D chemical structure of vemurafenib. (**B**) The 2D chemical structure of SJF-0628 consisting of vemurafenib, a short linker and a Von Hippel Lindau (VHL)-recruiting ligand. (**C**) The structure of BRAF-vemurafenib complex. BRAF kinase, ligand vemurafenib and identified pocket are colored in green, red and yellow, respectively. (**D**) The interaction between vemurafenib and BRAF kinase, in which the hydrogen bonds and other contacts are shown as blue and purple lines, respectively.

Proteins are vital biomolecules that play various biological roles in living organisms. All human life activities require proteins with indispensable functions, such as material transportation, catalysis, information exchange, immune function oxidation, functional maintenance and body acid–base balance. Without proteins, life would be impossible. However, protein cannot complete life activities independently. Instead, they must combine with other biomolecules like RNA, DNA, other proteins and organic and inorganic molecules, to accomplish specific functions [1–3]. For instance, most human kinases must bind to adenosine triphosphate (ATP) molecules to achieve various intracellular signaling and metabolic processes [4]. Mutations in human kinase protein residues may lead to abnormal kinase activity, causing hundreds of diseases, such as cancer and Parkinson’s [5–7]. Therefore, it is necessary to develop small organic molecules that target kinase proteins to regulate kinase activity and treat diseases [8–10]. This work will mainly focus on ligands (small organic molecules) as most drugs are organic small molecules. For example, vemurafenib and SJF-0628, as demonstrated in Figures 1A and B, are two inhibitors targeting human serine/threonine-protein kinase B-raf (BRAF). The former, an U.S. food and drug administration (FDA)-approved inhibitor, is widely used to treat late-stage or metastatic melanoma with BRAF V600E mutation [11]. The latter reduced the expression of all tested human BRAF mutants (V600E, K601E and G466E) but not the wild-type human BRAF in cell experiments [12].

The residues of proteins that interact with ligands are called binding sites or binding pockets [13, 14]. As shown in Figure 1C, the binding pocket refers to a cavity or groove on the surface of the target protein, where the ligand binds and interacts with the protein. Most small-molecule drugs exert their effects in the body through classic lock–key models, where drugs act as substrates and bind to the pockets, inhibiting or activating specific protein functions. Therefore, pockets have become a focus for researchers in molecular docking and drug design. In previous work, we established a human kinase drug pocket database to promote the development of specific drugs targeting human kinase proteins [15]. For ligands to achieve their functions, they must match their targeted pockets in space to avoid atomic space conflicts, and they should be tightly bound to the pocket with strong interactions to prevent the influence of thermal movement (Figure 1D). Drug molecules bind to active sites through hydrogen bonds, electrostatic interaction, hydrophobic interaction and relatively rare covalent bonds. The interaction strength of ligand binding to protein is quantified by binding affinity. The intrinsic activity of a drug refers to its ability to produce pharmacological effects after binding to its receptor. Drugs can be divided into two categories: agonists and antagonists [16–18]. Agonists are drugs with binding affinity and intrinsic activity, bind to receptors and stimulate them to produce effects. According to their intrinsic activity, agonists can be divided into complete agonists and partial agonists. The former has strong binding affinity and intrinsic activity, such as morphine. The latter has strong binding affinity but weak intrinsic activity, such as pentazosin. Antagonists bind to receptors with strong binding affinity but without intrinsic activity. They themselves do not have an effect, but exert antagonistic effects on agonists by occupying their binding sites, such as naloxone and propranolol. Both agonists and antagonists must be able to tightly bind to target proteins, meaning they must have high binding affinity. Therefore, the initial step in drug discovery is identifying small candidate ligands with high binding affinity that bind to the target protein, which can be further screened and optimized to identify lead compounds [19–21]. The protein–ligand binding affinity, thus, is one of the important indicators in drug screening.

In addition to the high binding affinity, the candidate ligands must bind to the target protein with high specificity, which means they should only target one or very few proteins to avoid attacking normal functional proteins, which may cause drug side effects [9, 22, 23]. To achieve this, it is necessary to calculate the binding affinity of thousands of ligands to a target protein and some candidate molecules’ binding affinity to multiple proteins when screening drugs. This process requires a robust computational workload. The binding affinity of the protein–ligand complex can be measured by experimental methods (such as isothermal titration calorimetry, surface plasmon resonance and methyl thiazolyl tetrazolium (MTT) assay) with various indicators such as inhibition constant (${K}_i$), dissociation constant (${K}_d$), Michaelis constant (${K}_m$), half-maximal inhibitory concentration (${IC}_{50}$) or median effective concentration (${EC}_{50}$) [24, 25]. The lower the ${K}_i$, ${K}_d$, ${K}_m$, ${IC}_{d50}$ and ${EC}_{50}$ values, the higher the protein–ligand binding affinities. However, these experimental methods are complex, expensive and time-consuming, which makes measuring the binding affinity of many different protein–ligand pairs a challenging process [26, 27].

The traditional methods used in computer-aided drug design methods, such as molecular docking and molecular dynamics simulation, evaluate protein–ligand binding affinity through scoring functions. Scoring functions used in molecular docking, such as AutoDock [28], X-Score [29] and ChemScore [30], are based on semi-flexible protein–ligand complex structures, which results in lower computational costs and computational accuracy. Scoring functions used in molecular dynamics simulation, such as Molecular Mechanics Poisson−Boltzmann Surface Area (MMPBSA), Molecular Mechanics Generalized Born Surface Area (MMGBSA) [31, 32] and Free energy perturbation [33], are mainly based on flexible protein–ligand complex structures and are more accurate but more computationally expensive. Usually, we need to optimize the structure of the target protein and provide a representative protein conformation before molecular docking. After the docking is completed, the accuracy of the protein–ligand complex obtained through the scoring function is not very high. In this case, dynamic simulation can calculate the binding energy and verify the effective binding between the protein and the screened small-molecule ligands. In addition, dynamic simulations can also be used to study the mechanism of action between drugs and target proteins, providing direct clues for drug design and the search for new drugs. Traditional scoring functions provide a theoretical basis for the binding affinity prediction and prompt the recognition of candidate molecules. There are over a hundred reported scoring functions, which are divided into three categories: force field, empirical and knowledge-based scoring functions [34–36]. However, these scoring functions are based on an incomplete physical model and approximated for simplified computation. Additionally, they heavily rely on manual planning and complex operations. Therefore, it is challenging for traditional calculation methods to accurately predict the binding affinities for numerous different protein–ligand pairs [37, 38].

Machine learning (ML) is a data-driven technology that uses statistics and algorithms to train models, enabling them to automatically discover patterns in data and make predictions. There are two types of ML models: traditional ML models and DL models. Traditional ML models, such as decision trees, support vector machines (SVMs) and random forests, have a simple model structure and low computational cost and are good at analyzing tasks with low dimensions and strong interpretability. For example, the RF-Score [39] uses protein–ligand atom-type pair counts on the binding site neighborhood as input for a random forest (RF) model to predict protein–ligand binding affinity. ID-Score [40] predicts protein–ligand binding affinity through an SVM model with 50 different protein–ligand binding descriptors. Research shows that the ML model has a low computational cost and strong interpretability, which is beneficial for explaining the importance of certain features in predicting protein–ligand binding affinity. However, traditional ML models require manual data processing and annotation, followed by extracting valuable features, which may lead to severe errors. For instance, RF-score has been proven ineffective in drug screening due to the oversimplified representation of protein–ligand complexes [41]. Moreover, predicting protein–ligand binding affinity is a highly complex process because it depends on the geometric shape, size, physicochemical properties of atomic residues and even complex elements such as other ligands of the target protein. Therefore, accurately predicting protein–ligand binding affinity is a challenge for traditional ML with simple structures and complex feature engineering.

DL models are a type of ML algorithm that use deep neural networks such as deep fully connected networks, deep convolutional neural networks (CNNs) and deep recurrent networks to learn patterns in data. Unlike traditional ML algorithms, DL models can automatically extract advanced features from raw data and perform feature representation and classification. When dealing with large amounts of data, DL outperforms traditional ML due to its ability to handle large-scale data. At present, the DL models are widely applied in various fields because of their strong ability in feature extraction and pattern recognition. In fact, the DL models have also broken through technological bottlenecks in some areas [42–44]. In bioinformatics, the Deepmind team proposed the AlphaFold2 method to predict protein structure based on the DL model, which significantly improved accuracy and disrupted the entire field of biology [45, 46]. Additionally, flexible docking is an unresolved bottleneck issue in predicting protein–RNA complex structures. To address this issue, Zeng *et al.* proposed DRPScore, a DL model, for identifying native-like RNA-protein structures [47]. The DRPScore can learn the interface features of protein–RNA complexes with fewer interaction data and has conducted extensive evaluations on test sets with different degrees of flexibility. The success rate of DRPScore in the rigid docking test set is 91.67%, while in the flexible docking test set is 56.14%, which is an increase of 10.53–15.79% compared to traditional methods. It is expected that DL models will play an increasingly important role in predicting protein–ligand binding affinity. Currently, there are numerous DL models for predicting protein–ligand binding affinity. However, it is still challenging for existing models to accurately predict protein–ligand binding affinity due to the low-quality database, inaccurate input representation and inappropriate model architecture, etc.

This paper is organized as follows. In sections Databases for  building the DL models to Models, we provide a detailed explanation of the different elements of DL-based methods that predict protein–ligand binding affinity. These include databases, input representations and models. In the final section, we summarized the existing challenges in developing DL models to accurately predict protein–ligand binding affinity and suggested future works for this rapidly expanding field.

## DATABASES FOR BUILDING THE DL MODELS

DL models extract features and learn intrinsic mechanisms by training large-scale data to solve complex problems. The database is divided into three parts: training set, validation set and testing set. The training set is used to fit and debug the network’s parameters for model learning. The validation set checks the training effect and determines whether the model training is progressing in a bad direction. If the model develops in a bad direction, training will stop on time and the model structure and hyperparameters can be adjusted accordingly, saving time. The validation set is unnecessary because it is only one means to help us find the optimal parameters. The test set is responsible for testing the network’s actual learning ability, evaluating the final model’s generalization ability, and not for parameter tuning. The training, validation and testing sets are the raw materials for model learning, optimization and evaluation, respectively.

The database quality directly affects the DL model’s prediction accuracy. High-quality databases can provide more accurate and comprehensive information, thereby improving the performance and generalization ability of the DL model. Therefore, the success of DL must be connected to a large number of high-quality databases. In this section, we give an overview of the data and the related databases. We studied 37 DL models (Table S1) published in the past 5 years. Thirty-three DL models developed for predicting protein–ligand binding affinity utilized the PDBbind database [48] as a training set, followed by the Davis database [49], the KIBA database [50] and the ChEMBL database [51]. The representative non-redundant subset for benchmark testing contains a core set of PDBbind database [48], Comparative Assessment of Scoring Functions (CASF) [52], Community Structure–Activity Resource (CSAR) [53], Astex Diverse set [54] and a ChEMBL bioactivity benchmark set [55]. These databases are summarized in Table 1 and can be divided into two categories: sequence data and structure data. Next, we will provide a detailed introduction to these representative databases.

**Table 1:** The representative databases for predicting protein–ligand binding affinity

Database	Training or test set	Type	Number of proteins	Number of ligands	Number of binding affinities	Given information
PDBbind-2020	Training set	Structure-based	19 433	19 433	19 433	Tertiary structures of protein pocket–ligand complexes
Davis	Training set	Sequence-based	442	68	30 056	Kinase protein sequences and ligand SMILES
KIBA	Training set	Sequence-based	229	2111	118 254	Protein sequences and ligand SMILES
ChEMBL v23	Training set	Structure-based	13,382	1 961 462	N.A.	2D structures of ligands
CASF-2016	Test set	Structure-based	N.A.	N.A.	285	Tertiary structures of protein pocket–ligand complexes
CSAR NRC-HiQ set 1	Test set	Structure-based	N.A.	N.A.	176	Tertiary structures of protein–ligand complexes
CSAR NRC-HiQ set 2	Test set	Structure-based	N.A.	N.A.	167	Tertiary structures of protein–ligand complexes
CSAR2012	Test set	Structure-based	N.A.	N.A.	57	Tertiary structures of protein–ligand complexes
CSAR2014	Test set	Structure-based	N.A.	N.A.	47	Tertiary structures of protein–ligand complexes
Astex Diverse set	Test set	Structure-based	N.A.	N.A.	74	Tertiary structures of protein–ligand complexes
ChEMBL bioactivity benchmark set	Test set	Structure-based	1227	204 085	314 767	2D structures of ligands

N.A., not applicable.

### PDBbind database

**Figure 2:**
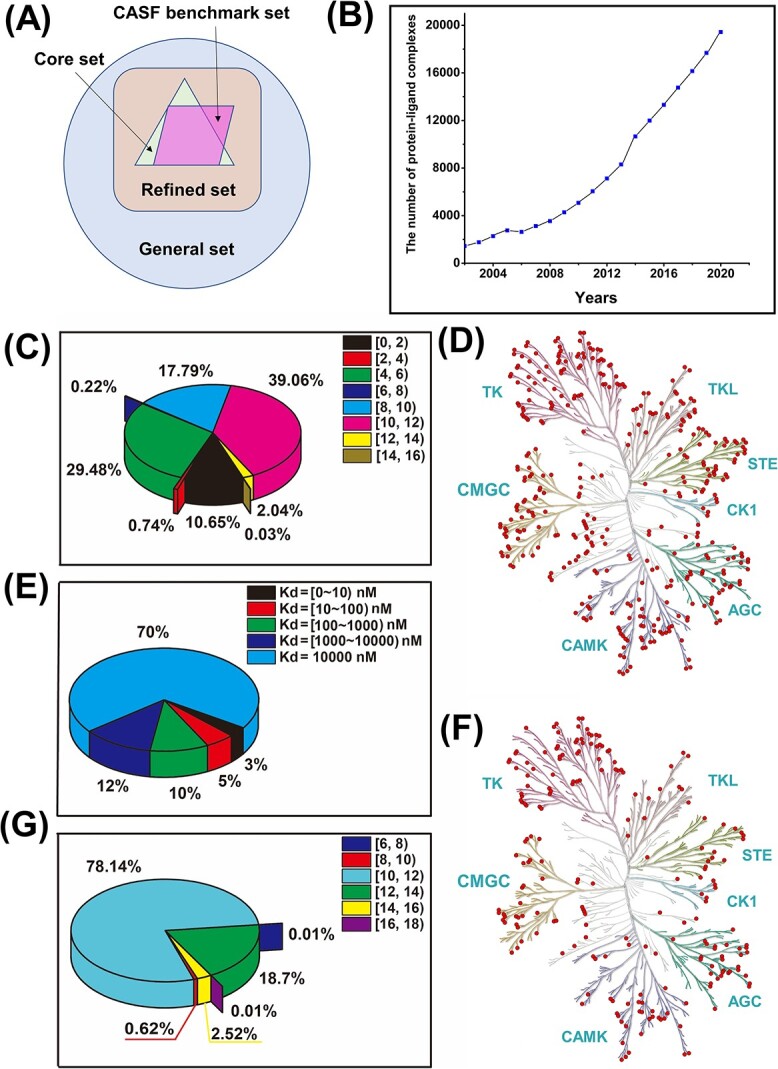
(**A**) Three overlapping subsets, including the general, refined and core sets, in the PDBbind database. (**B**) The number of protein–ligand complexes in the PDBbind database from 2002 to 2020. (**C**) The pie chart shows the distribution of protein–ligand binding affinity values in the PDBbind database. (**D**) The distribution of 367 human kinases in the Davis database on the human kinome tree in which the red dots represent each kinase. (**E**) The pie chart shows the distribution of protein–ligand binding affinity values in the Davis database. The lowest ${pK}_d$ values ($p{K}_d=5,{K}_d=10000\ nM$) constitute 70% of all binding affinity values in the Davis database. (**F**) The distribution of 216 human kinases in the KIBA database on the human kinome tree in which the red dots represent each kinase. (G) The pie chart shows the distribution of protein–ligand binding affinity values in the KIBA database.

The PDBbind database, first publicly released by the Shaomeng Wang group in 2004 [48], aims to comprehensively collect experimentally measured binding affinities for all biomolecular complexes in the Protein Data Bank [56]. The binding affinity values are expressed as ${pK}_a$ (−log${K}_d$, −log${K}_i$ or −log${IC}_{50}$). Figure 2C shows that the distribution of protein–ligand binding affinity values in the PDBbind database is relatively balanced. The binding affinity within the range of six to eight accounts for approximately 39% of the total sample size in the PDBbind dataset. For each protein–ligand complex, the tertiary structures of the pocket and its corresponding protein are provided in Protein Data Bank structure files (.pdb format). The pocket is composed of residues that are close to the ligand (less than 10 Å). Ligand structures are provided in biomolecular structure files (both .mol2 and .sdf formats). The MOL2 file is a chemical format file used to store molecular structure and related attribute information, which consists of atomic coordinates, bond connection information and other information metadata of molecules, such as charge and electronic state. These pieces of information can play an important role in molecular structure calculations and simulations. The SDF file is an extended version of a universal MOL file format, consisting of a series of MOL files and some additional information about compounds. The most significant difference is that MOL2 files contain structural information of individual molecular compounds in text format, while SDF files consist of a series of MOL files and some additional information about compounds in binary or American Standard Code for Information Interchange (ASCII) format. We can directly download the mol2 and SDF files of ligands in some small molecule databases, such as PubChem and BrugBank, or convert the PDB files of ligands downloaded from the Protein Data Bank into MOL2 and SDF formats through some software, such as OpenBable and RDkit.

The PDBbind database consists of three overlapping subsets: general set, refined set and core set (Figure 2A, Table S2). The general set includes all protein–ligand complexes in the PDBbind database. In contrast, the refined set, a subset of the general set, comprises protein–ligand complexes with higher-quality structures and binding affinity [57]. Specifically, the conditions to get the refined set are as follows: (1) The crystal structure resolution must be better than or equal to 2.5 Å. (2) The ligand must bind to the protein through non-covalent bonds to form binary complexes. (3) The binding affinity must be experimentally determined with a ${K}_i$ or ${K}_d$ value. (4) The protein and the ligand used in the binding assay must match the ones observed in the complex structure. (5) The ligand must be composed only of ordinary atoms, and its molecular weight should be lower than 1000. (6) There are no unnatural amino acid residues in the binding site on the protein. The core set is a non-redundant subset used for benchmark testing and includes representative protein–ligand complexes selected from the refined set by clustering with a 90% protein sequence similarity threshold. The number of complexes in the core set has fluctuated rather than constantly increased due to improved screening conditions for complexes (Table S2). For example, each cluster contains at least four members in the PDBbind-2010 database and at least five in the PDBbind-2011 database.

The research team of Professor Wang Renxiao is currently maintaining and developing the PDBbind database. The database has been updated annually since its inception, with the latest release being PDBbind-2020. The PDBbind-2021 is not a regular update but a major one, encompassing new binding data, new processed structures, new functions and a new cloud-based server. Table 2 provides basic information for each version of the database. Originally, the PDBbind database only contained protein–small ligand complexes, but in 2008, the protein–protein, protein–nucleic acid and nucleic acid–ligand were also added to the PDBbind database. As the number of Protein Data Bank entries gradually increases, the amount of protein–ligand complexes in the PDBbind database also gradually increases (Figure 2B). The DL model can discover patterns and rules in more data and better learn the essential laws of the problem, thereby more accurately predicting and classifying new data, improving the model’s generalization ability. It’s worth noting that version 2006 is a correction to the previously released version 2005.

**Table 2:** The basic information for each version of PDBbind database

Version	Entries In protein data bank	Valid biomolecular complexes	All complexes with binding affinity	Protein–ligand complexes	Protein–protein complexes	Protein–nucleic acid complexes	Nucleic acid–ligand complexes
2002	19 621	5671	1446	1446	N.A.	N.A.	N.A.
2003	23 790	5897	1763	1763	N.A.	N.A.	N.A.
2004	28 968	6847	2276	2276	N.A.	N.A.	N.A.
2005	34 338	9775	2756	2756	N.A.	N.A.	N.A.
2006	34 338	9775	2632	2632	N.A.	N.A.	N.A.
2007	40 876	11 822	3124	3124	N.A.	N.A.	N.A.
2008	48 092	18 211	4300	3539	471	250	39
2009	55 069	23 284	5678	4277	1053	304	44
2010	62 387	26 434	6772	5075	1281	361	55
2011	70 224	30 259	7986	6051	1441	428	66
2012	78 235	34 180	9308	7121	1597	511	79
2013	87 085	38 918	10 776	8302	1804	587	83
2014	96 952	44 569	12 995	10 656	1592	660	87
2015	105 183	48 821	14 620	11 987	1807	717	109
2016	114 344	53 838	16 179	13 308	1976	777	118
2017	124 962	59 805	17 900	14 761	2181	837	121
2018	135 859	65 851	19 588	16 151	2416	896	125
2019	146 836	71 897	21 382	17 679	2594	973	136
2020	157 974	78 460	23 496	19 433	2852	1052	149

N.A., not applicable.

### Davis database

The Davis database [49] is a large-scale dataset from selectivity assays of the kinase proteins and the clinically relevant inhibitors with their respective ${K}_d$ values. It comprises 442 kinase proteins and 68 ligands with 30 056 binding affinity values. The ligand SMILES and protein sequence are provided in text files (.txt format). The binding affinity values are usually quantified using $p{K}_d$ values, as explained in Equation (1).


(1) \begin{equation*} p{K}_d=-\mathit{\log}10\left(\frac{K_d}{1e9}\right) \end{equation*}

There are 367 human kinases out of 442 proteins in the Davis database. Figure 2D shows the uniform distribution of 367 human kinases in the human kinome tree. The value range of ${K}_d$ is 0.01 to 10 000 nM, and the corresponding binding affinity value range is 5–11 according to Equation (1). The lowest $p{K}_d$ values ($p{K}_d=5$) corresponding to the negative protein–ligand complexes that either interact with weak binding affinities or are not observed in the experiment for selectivity assays, constitute 70% of all binding affinity values in the Davis database (Figure 2E).

**Figure 3:**
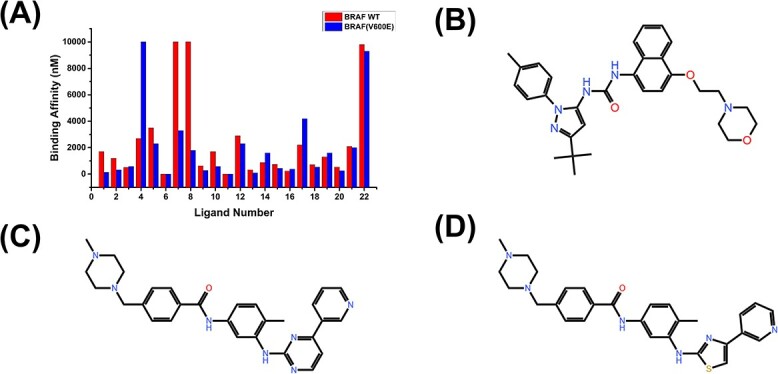
(**A**) The binding affinity values of 22 ligands in the Davis database targeting wild-type BRAF and BRAF V600E mutant. (**B**–**D**) The structure diagrams of the 4th, 7th and 8th ligands in Figure 3A, respectively.

Kinase protein residue mutations are one of the leading causes of diseases such as cancer. For instance, the V600E mutation in the BRAF human kinase is a common mutation in many malignant tumors and is often considered a marker of poor prognosis. It can lead to abnormal activity in cell division, proliferation, differentiation and other aspects, thereby promoting the growth and spread of cancer cells. The BRAF V600E mutation is common in thyroid cancer, ovarian cancer, etc. Among 68 ligands in the Davis database, 22 ligands target wild-type BRAF and/or BRAF V600E mutants with different binding affinities, as shown in Figure 3A. The result shows that the 4th ligand specifically targets the wild-type BRAF, while the 7th and 8th ligands specifically target the BRAF V600E mutation. The molecular structure diagrams of these three ligands are shown in Figures 3B–D, respectively.

### KIBA database

The KIBA database originated from a KIBA score function constructed to optimize the consistency between ${K}_d$, ${K}_i$ and ${IC}_{50}$ [50]. The binding affinity values were measured by KIBA scores ranging from 0 to 18. Initially, the KIBA database contains 467 kinase proteins and 52 498 ligands. He *et al.* further screened protein–ligand pairs with at least 10 binding affinities based on the KIBA database, yielding 229 unique kinase proteins and 2111 unique ligands with 118 254 binding affinities in the KIBA benchmark database [58]. The ligand SMILES and protein sequence are provided in TXT formats. Figure 2F shows the uniform distribution of 216 human kinases in the human kinome tree. However, the binding affinity distribution is uneven (Figure 2G). The binding affinity within the range of 10–12 accounts for approximately 80% of the total sample size in the KIBA dataset.

### ChEMBL database

ChEMBL is an extensive, open-access drug discovery database that contains 13 382 target proteins and 1 961 462 different compounds in the 23rd version. The ChEMBL database provides ligand SMILES and corresponding target ChEMBL ID. To build a reliable training dataset for classification prediction, Wang *et al.* filtered and preprocessed the data based on different types and bioactivity measurements [59]. For each target protein, ligands with bioactivity values ≤10 μM are selected as positive samples, while ligands with bioactivity values ≥20 μM are selected as negative samples, resulting in a much larger number of positive samples of each target protein than negative samples. Wang *et al.* populated the negative training sets by a target similarity–based inactive dataset enrichment method to balance the number of positive and negative samples [59]. Finally, the number of positive and negative samples, with a ratio of approximately 1:1, is 475 238 and 430 590, respectively.

### CASF database

The CASF database, as a mainstream open-access Comparative Assessment of Scoring Functions benchmark set, is often used to evaluate the prediction accuracy of DL models for predicting protein–ligand binding affinity. There are three versions of the CASF benchmark set: CASF-2007, CASF-2013 and CASF-2016. The CASF-2007 benchmark set (PDBbind-2007.2 core set) contains 195 protein–ligand complexes from 65 clusters. The CASF-2013 benchmark set directory contains the structural files of the 195 protein–ligand complexes in the PDBbind-2013 core set. It is worth noting that the CASF-2016 benchmark set contains 285 protein–ligand complexes while the PDBbind-2016 core set contains 290 protein–ligand complexes, in which 284 protein–ligand complexes coexist in the CASF-2016 benchmark and PDBbind-2016 core set [60–63].

### CSAR database

The CSAR database provided crystal structures and binding affinities (${K}_d$) for diverse protein–ligand complexes in .pdb/.mol2 and .dat formats, respectively [53]. The CSAR contains four well-known subsets (CSAR NRC-HiQ data set 1, CSAR NRC-HiQ data set 2, CSAR2012 and CSAR2014) that are popular as test sets of models for predicting protein–ligand binding affinity. Two CSAR NRC-HiQ subsets composed of 176 and 167 protein−ligand complexes were extracted from the BindingMOAD43 database and previous iterations of the PDBbind data set. The CSAR2012 and CSAR2014 were constructed with 57 and 47 protein–ligand complexes, respectively.

### Astex diverse set

The Astex diverse set [54] comprises 85 protein–ligand complexes with 11 complexes without binding affinity. The remaining 74 binding affinity values were measured in terms of the ${K}_d$, ${K}_i$, ${K}_m$ or ${IC}_{50}$. Notably, the proteins in these 85 complexes belong to different protein classes, including cyclin-dependent kinase 2, penicillin G acylase and deoxyhemoglobin.

### ChEMBL bioactivity benchmark set

The ChEMBL bioactivity benchmark set [55] was obtained from ChEMBL.v20 according to the following conditions: (1) each target protein contains at least 30 compounds from at least two separate publications and the assay confidence score of 9; (2) binding affinity values with activity comments ‘not active’, ‘inactive’, ‘inconclusive’ and ‘undetermined’ were removed; (3) If multiple measurements for a ligand–protein binding affinity were present, the median value was chosen and duplicates were removed. Finally, the ChEMBL bioactivity benchmark set consisted of 1227 targets and 204 085 compounds with 314 767 binding affinity values. The data files can be downloaded from https://data.4tu.nl/datasets/51a90ed0-9f8a-46fc-9497-d44aeced28ed/1.

## INPUT REPRESENTATION

The feature and representation of the protein and ligand are crucial for the prediction performance of DL models. Some models use protein monomers and ligand monomers as inputs to the models, while others use protein–ligand complexes as inputs. The former does not require a known protein–ligand binding posture, so its applicability is wider. The latter contains direct information on protein–ligand interactions, resulting in higher prediction accuracy. This section summarizes the input representations of protein, ligand and protein–ligand complex, including both sequence-based and structure-based input representations.

### Ligand input representation

#### Two-dimensional structure–based input representation for ligand

Some databases, such as Davis [49] and KIBA [50], provide ligand SMILES string that uses a sequence of characters to describe ligand Two-dimensional (2D) structure based on atoms, bonds, rings, etc. These ligand SMILES strings were typically encoded by integers as input features. For example, Öztürk *et al.* scanned approximately 2 M ligand SMILES and compiled 64 characters encoded as 1–64 [64]. To ensure that all ligand SMILES are encoded with the same length, ligand SMILES were truncated or padded with 0 s to the allowed maximum length L. In addition, the ligand SMILES can be further converted to a 2D matrix or a 2D molecular graph [65–67].

#### Bioactive features of ligand

Bioactive properties of ligands play a key role in predicting protein–ligand binding affinity, which was extracted from the Chemical Checker data set based on ligand InChIKey. The Chemical Checker data set provided five levels of bioactivity data, including chemistry, targets, networks, cells and clinics on ∼800 000 ligands in a vector format [65].

### Protein input representation

#### Sequence-based input representation for protein

Generally, protein sequences consist of amino acids encoded by integers or one-hot vectors based on amino acid types as input features [64, 68]. In recent years, some features, such as secondary structure, position-specific scoring, relative solvent accessibility and dihedral angles, were predicted by existing calculation methods based on protein sequences [68, 69].

#### Structure-based input representation for protein

Researchers often use local pocket structures instead of global protein structures as inputs based on two facts: (1) because of the large size of the protein, the cost of DL models is huge to directly encode the global protein structures as inputs and (2) ligand directly binding to a local pocket that refers to a protein interior or surface cavity. The condition for stable binding between ligands and pockets is that they have a matching geometric shape, and the atoms of the pockets have strong intermolecular interactions with the atoms of the ligands. Therefore, the pocket information, including sequences, secondary structure elements, tertiary structures and physicochemical characteristics, are usually represented as three-dimensional (3D) grid, implicit graphs or point cloud and then used as the input features in some models to predict protein–ligand binding affinity [67–69].

### Protein–ligand complex input representation

#### Local structure

The protein–ligand binding affinity mainly depends on the interaction between the ligand and protein pocket. The pocket–ligand complex structure contains most key interactions between protein and ligand. It can avoid computational complexity caused by the large global protein structure, making it a more common input sample. Jiménez *et al.* [70] defined the pocket as a local structure composed of protein residues in a 24 Å^3^ grid centered around the geometric center of the ligand. The pocket–ligand complex was represented by a 3D voxel representation based on a van der Waals radius for each atom type and some physicochemical properties, including hydrophobicity, aromaticity and iconicity. Stepniewska-Dziubinska *et al.* [37] selected the local structure of a protein with a 20 Å cubic box focused at the geometric center of the ligand as a pocket. The pocket–ligand complex structure is represented by a four-dimensional tensor based on Cartesian coordinates and 19 features of atoms [37]. The 19 features represent atom information, including atom types, atom hybridization, the number of bonds, SMARTS (SMiles ARbitrary Target Specification) patterns and partial charge. Rezaei *et al.* [71] selected a 32 Å cubic box according to end-to-end distribution for all ligands in the PDBbind v.2016. They represented nine major heavy atoms (C, N, O, P, S, F, Cl, Br, I) with excluded volume features and 11 atom types. Karlov *et al.* [72] developed the graphDelta model based on atom types and atomic environment in binding sites. The descriptors of the atomic environment depend only on the distance to the neighboring atoms and the angles formed by all-atom pairs in the environment and the central atom. Zhang *et al*. developed the DeepBindRG model [73] that implicitly learns all the effects, binding mode and specificity by protein–ligand interface contact information. A 2D interaction feature map of 1000*125 represents the interface contact information. Each line represents a pair of interacting atoms between a protein–ligand complex in which the ligand and protein atom types are encoded by 84-dimension and 41-dimension one-hot representation, respectively. The maximum line number is defined as 1000, which covers almost all of the pair numbers. Xia *et al*. [74] constructed the ligand, protein and protein–ligand interaction graphs based on atomic position, bound and atomic distance as inputs of a graph neural network (GNN) model. In this model, only the local protein structure close to the ligand is used to construct the protein and protein–ligand interaction graphs.

#### Global structure

The cost of DL models is huge to encode the complete global protein–ligand complex structures as inputs directly. However, the long-range interactive information between the protein and the ligand will be lost if only local structures are considered. To overcome the above limitation, researchers extract interaction information from the global structure of the protein–ligand complex as inputs to the model. Zheng *et al.* [75] extracted the intermolecular interaction information from the global tertiary structures of protein–ligand complexes. They defined the input features of the OnionNet model based on rotation-free element-pair-specific contacts between ligands and protein atoms. The contacts cover both the local and non-local interaction. Moon *et al.* [76] developed a PIGNet model that takes molecular graphs and distance information of protein–ligand complexes as inputs. The molecular graph comprises intermolecular and intramolecular interactions between protein and ligand. The distances between the atom pairs of a protein–ligand complex compensate for the limitation of a molecular graph lacking spatial information.

### Environment element input representation

In addition to protein and ligand molecules, some environmental factors have also been introduced as inputs. For example, Qu *et al.* introduced the water network to characterize the protein–water interactions and protein–ligand–water interaction [77].

## MODELS

**Figure 4:**
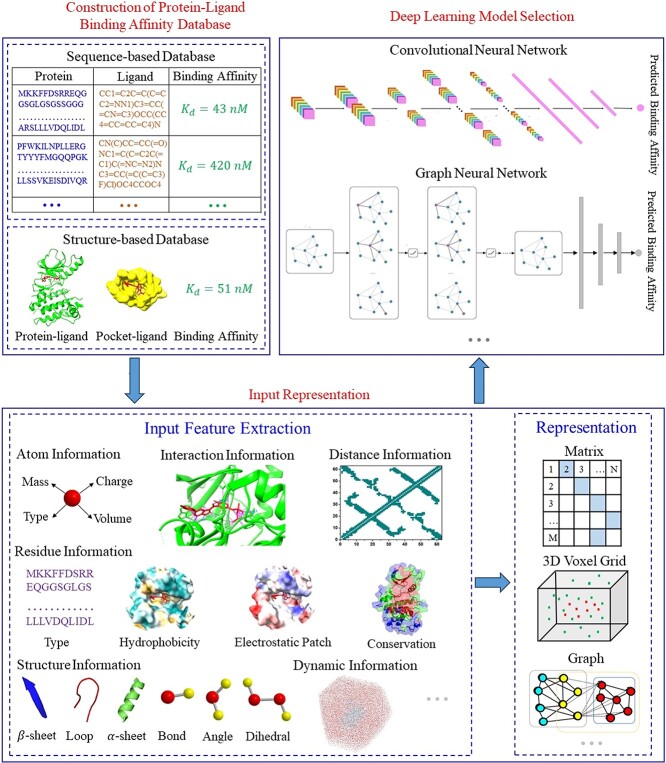
An overall flowchart for predicting protein–ligand interactions based on DL models.

**Figure 5:**
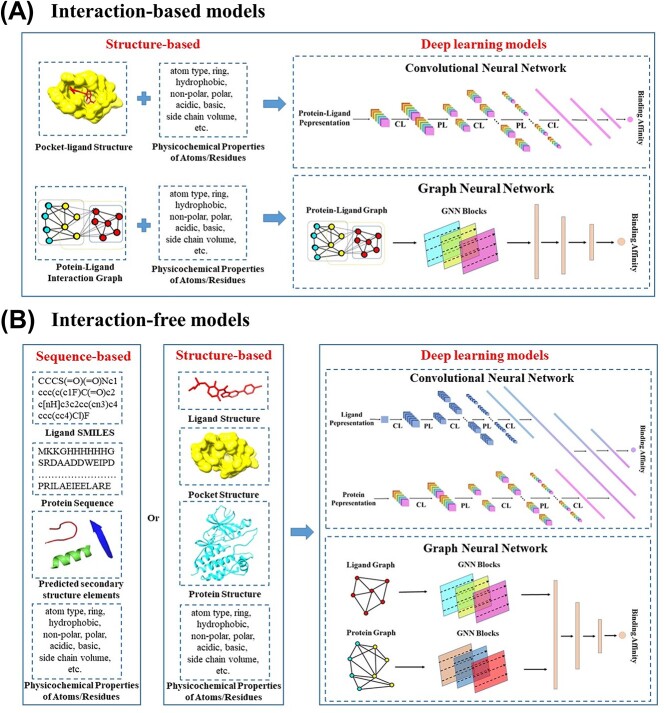
(**A**) Conceptual workflow of interaction-based DL models. Inputs are the pocket–ligand complex structures and their characteristics. (**B**) Conceptual workflow of interaction-free DL models. The structure-free models can predict protein–ligand binding affinity without protein–ligand interaction information. The inputs of interaction-free models are ligand SMILES strings/protein sequences or ligand/protein monomers 3D structures and their characteristics.

DL models have become increasingly popular in bioinformatics due to their ability to extract advanced features and identify functional patterns from raw data. Table S1 summarizes the DL models published in the past 5 years for predicting protein–ligand binding affinity. Three crucial steps in predicting protein–ligand binding affinity via the DL models are shown in Figure 4. Firstly, a rich protein–ligand binding affinity database, which includes a large number of training samples, is essential. Secondly, effective input features should be extracted from training samples and characterized as inputs to DL models. Lastly, selecting a suitable DL model is crucial to accurately predicting protein–ligand binding affinity. These models can be divided into two categories, interaction-based and interaction-free models, based on whether the input features of models contain protein–ligand interaction information (Figure 5, Table S1).

Interaction-based models extract information about protein–ligand interactions from the 3D structures of protein–ligand complexes, which have been demonstrated to play an essential role in improving the model’s generalization capability (Figure 5A). Twenty-nine out of the 37 DL models in Table S1 belong to interaction-based models. Some models, such as K_DEEP_ [70], pafnucy [37], DeepAtom [71] and AK-score [78], take a 3D voxel grid to represent the 3D structures of protein–ligand complexes as input features of CNNs. The different atom information (hydrogen bond, hydrophobicity, aromaticity, excluded volume, etc.) is encoded into different channels of the 3D grid. These models aim to learn the spatial distribution and feature information of atoms in the 3D voxel grid by CNNs, thereby obtaining protein–ligand interactions such as hydrogen bond and hydrophobic interactions and ultimately predicting protein–ligand binding affinity. However, the 3D voxel grid representation has several limitations. First, these grid sizes are usually defined around a protein’s binding site due to the limitation of computational cost, resulting in the absence of long-range interactions. Second, the CNNs use 3D convolutions to extract interaction information from a 3D voxel grid with lower efficiency, as most voxels do not contain useful topology information. Third, limited atomic features are insufficient to provide all non-covalent interactions, which are crucial for predicting protein–ligand binding affinity. In addition, the sensitivity of 3D grids to rotation has a negative impact on the prediction results. Other models, including OnionNet [75], Fast [79] and SIGN [80], are based on contacts between protein and ligand atoms with a matrix or a 2D graph representation to predict protein–ligand binding affinity. For example, the OnionNet model takes the contacts between protein and ligand atoms as the input feature of a CNN model. The contacts are grouped into different distance ranges to cover both the short-range and long-range interactions between protein and ligand. However, interaction-based models are limited to known protein–ligand complex structures.

In contrast, interaction-free models assume that DL models can predict protein–ligand binding affinity without protein–ligand interaction information. The inputs of interaction-free models include ligand SMILES strings/protein sequences and ligand/protein monomers structures (Figure 5B). DeepDTA, an interaction-free model, uses two CNN blocks to extract ligand and protein features from ligand SMILES strings and protein sequences, respectively [64]. Similarly, Limbu *et al.* developed a hybrid neural network–affinity model based on only ligand SMILES strings and protein sequences as input features [66]. In addition to the ligand SMILES strings and protein sequences, the interaction-free models, such as DeepDTAF [64], DEELIG [69], PLA-MoRe [65], PLANET [67] and CAPLA [81], also take the pocket sequence, the predicted secondary structure, atomic and residual physicochemical properties of protein/pocket, as well as the bioactive properties of the ligand as inputs. Although the interaction-free models are not limited to known protein–ligand complex structures, they still have the following limitations. Sequence-based models require a high homology of samples. The samples in both existing sequence-based databases are kinase–ligand pairs. Additionally, the inputs of sequence-based models are generally preprocessed by other computational methods, such as multi-sequence alignment and secondary structure prediction methods. The computational accuracy of these methods can affect the prediction accuracy of the DL models. Furthermore, the inputs of the interaction-free model lack effective interaction, topology and chemical bond information, which makes it difficult to accurately predict protein–ligand binding affinity.

In addition, existing DL models predicting protein–ligand binding affinity are mainly based on CNNs and GNNs. A CNN is a neural network structure specifically designed for image and other types of multidimensional data processing. The CNN typically comprises convolution, pooling and fully connected layers. The convolution layer uses convolutional verification to perform convolutional operations on the input image, extract local features and improve the model’s effectiveness. The convolution layer in CNN usually contains multiple convolution kernels, each of which can extract different features from the image. Convolutional computation is an important operation in CNNs, which extracts features by sliding a convolutional kernel on input data. The following is the formula for convolution calculation:


(2) \begin{equation*} \mathrm{y}\left[i,j\right]={\sum}_{m=-\frac{k}{2}}^{\frac{k}{2}}{\sum}_{m=-\frac{k}{2}}^{\frac{k}{2}}x\left[i+m,j+n\right]\cdot w\left[m,n\right] \end{equation*}

where $x$ is the input data, $y$ is the output of the convolution calculation, $w$ is the convolution kernel, $k$ is the size of the convolution kernel, the subscripts $i$ and $j$ represent the position of the output and $m$ and $n$ represent the position of the convolution kernel. The pooling layer reduces the dimensionality of the feature map through down-sample operations while retaining critical information and preventing overfitting. The fully connected layer converts the feature map into a one-dimensional (1D) vector and calculates the predicted results through weight calculation. CNN models’ most powerful advantage is their ability to capture local features through convolution kernels.

The existing CNN models for predicting protein–ligand binding affinity are divided into 1D and 3D CNNs. The 1D CNNs are usually interaction-free models and comprise two or more convolutional blocks, mainly taking sequence information as inputs. For example, the DeepDTA model includes two separate CNN blocks that learn representations from protein sequences and ligand SMILES strings, respectively [64]. Based on the DeepDTA, Wang *et al.* developed the DeepDTAF model, including three separate CNN blocks that take global protein, local pocket and ligand features as inputs, respectively. Compared to DeepDTA, the DeepDTAF model incorporates secondary structural information and protein and pocket physicochemical characteristics. The DeepDTAF model outperformed DeepDTA for the core 2016 test set of the PDBbind database, test105 set and test71 set collected from the Protein Data Bank in terms of root mean squared error (RMSE), mean absolute error (MAE), Pearson’s correlation coefficient (R), standard deviation (SD) and concordance index (CI), respectively. The 3D CNNs, such as pafnucy [37], are usually interaction-based models and include one convolutional block. The convolutional block takes a cubic grid box that represents the atom’s coordinates of a pocket-ligand complex as input. However, CNN models have good computational complexity and generalization ability. It is a massive cost for 3D CNN models to consider long-range interactions. In addition, the absence of explicit interaction representation and the sensitivity to rotation in the complex may limit 3D CNN models. Based on the advantages of 1D and 3D CNN models, we developed the DLSSAffinity model by combining DeepDTA and pafnucy [82]. The DLSSAffinity model comprises two 1D CNN blocks and one 3D CNN block. One of the 1D CNN blocks is responsible for learning long-range interactions from protein sequences. In contrast, the 3D CNN module is accountable for learning short-range interactions from pocket–ligand complex structures. The results indicate that the DLSSAffinity model outperforms DeepDTA and pafnucy.

With the advancements in ML, significant breakthroughs have been made in speech, image and natural language processing, all of which are simple sequence or grid data. However, not all real-world phenomena can be represented as a sequence or a grid, such as social networks, protein interactions and protein–ligand interactions. Compared to simple text and images, a network graph is complex and poses difficulties in processing, including the following: (1) graph size is arbitrary; (2) graph topological structure is complex and lacks spatial locality; (3) the graph does not have a fixed node order; and (4) graphs are often dynamic. These issues gave rise to GNNs, which embed nodes based on their local neighbor information. In simple terms, GNNs aggregate the information of each node and its neighboring nodes through a neural network. In the following sections, we will briefly introduce the basic concepts of graphs, their representations and the classic GNN model.

The graph $G\left(V,E\right)$ is a data structure containing a set of nodes ${v}_i\in V$ and a set of edges ${e}_{ij}=\left({v}_i,{v}_j\right)\in E$ connecting nodes ${v}_i$ and ${v}_j$. In GNNs, common methods for representing graphs include the adjacency matrix, degree matrix, Laplacian matrix, etc. The adjacency matrix is used to represent the relationships between nodes in a graph. For a simple graph with *n* nodes, there is an adjacency matrix $A\in{\mathrm{R}}^{n\times n}$


(3) \begin{equation*} {A}_{ij}=\left\{\begin{array}{@{}cc@{}}1,&\left({e}_{ij}\in E\ and\ i\ne j\right)\\{}0,& else\ \end{array}\right\} \end{equation*}

The degree of a node ${v}_i$ represents the number of edges connected to it, denoted as $d(v)$. For a simple graph $G\left(V,E\right)$ with $n$ nodes, its degree matrix $D$ is ${D}_{ii}=d(v)={\sum}_j{A}_{ij}$, which is a diagonal matrix. The laplace matrix is defined as $L=D-A$. After normalization,


(4) \begin{equation*} {L}^{sym}={D}^{-1/2}L{D}^{-1/2} \end{equation*}

The node ${v}_i$ in the graph has a feature vector, which is saved using matrix $X$. The ${x}_i\in X$ represents the feature vector of the node ${v}_i$. Unlike other neural networks, the input of GNNs is a graph, and calculations are performed on the graphs to obtain outputs that are also graphs. The GNNs have many models, mainly divided into the graph convolution network (GCN), graph attention network, graph auto-encoder, graph generative network and graph spatial–temporal network. A detailed introduction to the GNNs can be found in the review literature [83]. Here, we will briefly introduce the GCN, which is the foundation of many complex GNN models. The convolution of a pixel in an image is a weighted sum of that pixel and its neighboring pixels. Therefore, in the graph structure, the convolution of a node should be a weighted sum of that node and its neighboring nodes. In 2017, Kipf *et al.* [84] proposed a two-layer GCN with activation functions of ReLU and Softmax, respectively. The formula for forward propagation is as follows:


(5) \begin{equation*} Z=f\left(X,A\right)= softmax\left(\hat{A}\ ReLU\left(\hat{A}X{W}^{(0)}\right){W}^{(1)}\right) \end{equation*}

(6) \begin{equation*} \hat{A}={\tilde{D}}^{-1/2}\tilde{A}{\tilde{D}}^{-1/2} \end{equation*}

where ${W}^{(0)}$ and ${W}^{(1)}$ represent the weight matrices of the two layers. $\tilde{A}=A+I$. The $I$ is an identity matrix. The transfer formula for GCN is


(7) \begin{equation*} {H}^{\left(l+1\right)}=\sigma \left({\tilde{D}}^{-1/2}\tilde{A}{\tilde{D}}^{-1/2}{H}^{(l)}{W}^{(l)}\right) \end{equation*}

where ${H}^{(l)}$ represents the feature vector of the $l$-th layer of the network, The $\sigma$ represents a non-linear activation function. The product of the normalized matrix and the eigenvector matrix is as follows:


(8) \begin{equation*} \left({\tilde{D}}^{-\frac{1}{2}}\tilde{A}{\tilde{D}}^{-\frac{1}{2}}H\right)=\left({\tilde{D}}^{-\frac{1}{2}}\tilde{A}\right){\tilde{D}}^{-\frac{1}{2}}H=\left(\sum_k{\tilde{D}}_{ik}^{-\frac{1}{2}}{\tilde{A}}_i\right){\tilde{D}}^{-\frac{1}{2}}H \end{equation*}

(9) \begin{equation*} {\tilde{D}}_{ii}^{-\frac{1}{2}}\sum_j{\tilde{A}}_{ij}\sum_k{\tilde{D}}_{jk}^{-\frac{1}{2}}{H}_j={\tilde{D}}_{ii}^{-\frac{1}{2}}\sum_k{\tilde{A}}_{ij}{\tilde{D}}_{jj}^{-\frac{1}{2}}{H}_j=\sum_j\frac{1}{\sqrt{{\tilde{D}}_{ii}{\tilde{D}}_{jj}}}{\tilde{A}}_{ij}{H}_j \end{equation*}

From the above equation, it can be found that interlayer propagation not only takes the average of the features of neighboring nodes but also considers the degrees of node $i$ and its adjacent node $j$ by introducing the degree matrix D to normalize the adjacency matrix in GCN. This means nodes with higher degrees will contribute less during aggregation. Kipf *et al.* have demonstrated the excellent performance of GCN through experiments [84].

GNN models for predicting protein–ligand binding affinity can be classified into interaction-based and interaction-free models. Interaction-free GNN models like PLANET [67] transform protein and ligand structures into two separate graphs, where bonds and distance between atoms define the edges, and the nodes denote the amino acids in the protein and atoms in the ligand. Interaction-based GNN models like SIGN [80], GraphscoreDTA [85], EGNA [74] and GIGN [86] construct a graph to represent protein–ligand interaction information as input. GNN models are invariant to rotations, which makes their graph representations more robust than grid representations. However, most of the existing GNN models for predicting protein–ligand binding affinity aim to learn the spatial structure based on distance information, which can limit the prediction accuracy for binding affinity of larger protein–ligand complexes. Furthermore, the GNN model’s ability to capture fundamental long-range interaction information between protein and ligand is limited, which is valuable for predicting protein–ligand binding affinity.

## CHALLENGES AND FUTURE DIRECTION

Determining protein–ligand binding affinity through computational methods can promote drug discovery and development. In the past 5 years, many DL models have been proposed to predict the binding affinity between proteins and ligands, and significant performance improvements have been achieved. However, predicting the binding affinity between proteins and ligands still faces many challenges.

There are four challenges related to databases. Firstly, sequence-based databases, such as the Davis and KIBA databases, provide tens of thousands of kinase protein–ligand binding affinity values, which seems sufficient to train a DL model. However, these databases only contain hundreds of proteins or even dozens of ligands, which can cause an underfitting state and poor learning outcomes. Secondly, there is often a severe data imbalance in the sequence-based datasets. The negative protein–ligand complexes account for 70% of the total sample size in the Davis dataset. The binding affinity within the range of 10–12 accounts for approximately 80% of the total sample size in the KIBA dataset. It has been found that unbalanced samples in training sets often result in poor performance. Thirdly, there is a need to develop a sequence-based database for other proteins, as both existing large sequence-based databases are related to kinase proteins only. Fourthly, the sample size of structure-based databases is limited by the quantity and quality of existing crystal structures of protein–ligand complexes in the Protein Data Bank. Therefore, there is an urgent need to develop a sequence-based database with balanced samples or to find effective methods to solve the issue of unbalanced samples. In addition, developing a sequence-based database for other proteins is also a meaningful task.

In addition, the construction conditions of a database are also worth our attention, as they determine the quality of the database and lead to the emergence of multiple databases. For example, a given complex may have multiple binding affinities due to different experimental conditions, such as pH value, temperature and cell type. Each protein–ligand pair in the KIBA database has at least 10 binding affinity values to address this issue. A score function was constructed to optimize the consistency between ${K}_d$, ${K}_i$ and ${IC}_{50}$, thereby obtaining a KIBA score as protein–ligand binding affinity. Unlike the KIBA database, the protein–ligand binding affinities in the Davis database were obtained under the same experimental conditions, which results in low applicability and fewer sample sizes. Therefore, the KIBA database is currently the best sequence-based database. The PDBbind database is currently the most widely used due to its large sample size and strict filtering conditions.

In addition to high-quality databases with multiple samples, an accurate and reasonable input representation is also one of the conditions for improving the prediction accuracy of DL models. In recent years, researchers have incorporated the characteristics of residues (such as non-polar, polar, acidic, basic and side chain volume) and predicted secondary structures as input features for sequence-based models, improving the model’s prediction accuracy. However, the existing input representation methods for sequence information still face challenges in characterizing the impact of mutated residues on protein–ligand binding affinity. It is worth noting that Mahmud *et al.* recently proposed the PreMut method, which is based on equivariant GNNs and can accurately predict protein tertiary structural changes caused by single-point mutations [87]. The existing results indicate that the PreMut method has better accuracy than AlphaFold in predicting the tertiary structure of single-point mutant proteins. In the future, sequence-based DL models can attempt to introduce predicted mutated protein structures as inputs to improve the accuracy of DL models in predicting mutated protein–ligand binding affinity. Moreover, regarding the different lengths of protein sequences, we can unify the length of all sequences through multiple sequence alignment, which may be better than simply using length truncation, as the proteins in the Davis and KIBA database belong to the same class of proteins (kinase proteins) and have high homology.

**Figure 6:**
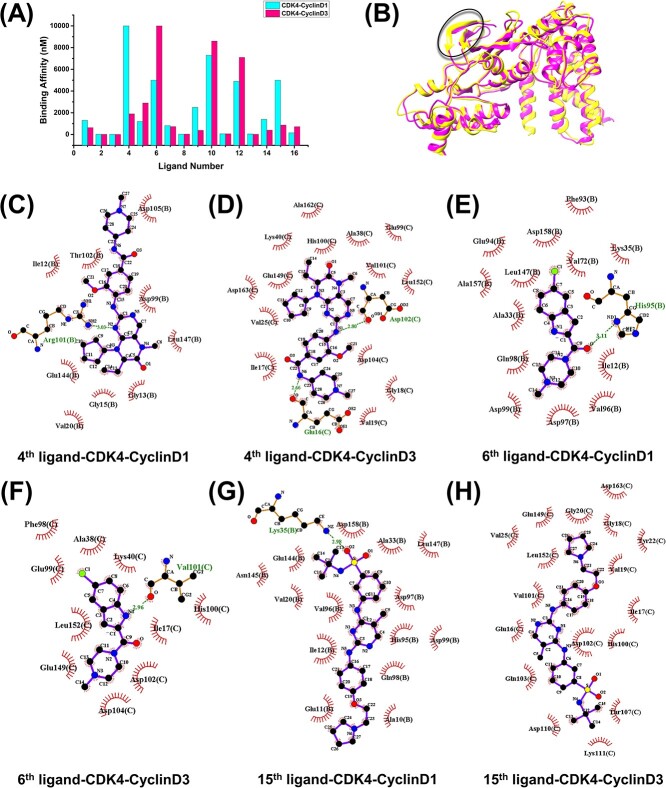
(**A**) The binding affinity values of 16 ligands in the Davis database targeting CDK4-CyclinD1 and CDK4-CyclinD3 complexes. (**B**) The structures of CDK4-CyclinD1 and CDK4-CyclinD3 complexes. (**C**–**H**) Diagrams of 2D protein–ligand interaction for the 4th, 6th and 15th ligands targeting CDK4-CyclinD1 and CDK4-CyclinD3 complexes in Figure 6A, respectively. The hydrophobic residues of protein and hydrophobic interactions between residues and ligands are colored in red. The hydrogen bonds between residues and ligands are indicated by the green lines. The hydrogen bond residues are colored in yellow and names are colored in green.

Structural information, especially the protein–ligand complex structure, implies more interaction information between protein and ligand, which is critical for a DL model to predict protein–ligand binding affinity accurately. The most popular input representations for structure-based models are 3D voxel grids of pocket–ligand pairs for CNN models and interaction graphs of protein–ligand pairs for GNN. Both 3D voxel grids and interaction graphs combined the physicochemical properties of atoms/residues. However, two challenges to the input representation feature of structure-based models still need to be addressed. Firstly, existing research suggests that the binding affinity of a given protein–ligand pair may vary in different cells due to different cell environments and different protein substrates for a given target protein. As shown in Figure 6A, 16 out of 68 ligands in the Davis database, primarily the 4th, 6th and 15th ligands, bind to the CDK4-CyclinD1 and CDK4-CyclinD3 complexes with different binding affinities. These phenomena are caused by the different structures of CDK4, especially the β-sheets in the catalytic domain (Figure 6B). The interaction for three ligands targeting CDK4-CyclinD1 and CDK4-CyclinD3 complexes is shown in Figures 6C–H. The results indicate that the interactions between the 4th/15th ligands and CDK4-CyclinD3 are stronger than those with CDK4-CyclinD1, while the interaction between the sixth ligand and CDK4-CyclinD1 is stronger than that with CDK4-CyclinD3. These conclusions are consistent with the binding affinity results measured in the experiment. However, the existing inputs lack cellular environmental information and substrates binding to target proteins. Second, ligand binding to protein is a dynamic process. The changes in atomic position and binding posture can provide important information for understanding ligand binding target proteins. Therefore, the dynamic structural information is crucial for accurately predicting protein–ligand binding affinity. Wu *et al.* proposed the ProtMD method, which has two types of self-supervised learning tasks, atomic level and conformational level, to better capture the internal and global information of molecular dynamics trajectories [88]. Compared with the most commonly used method, the ProtMD method has made significant improvements, reducing the RMSE of protein–ligand binding affinity issues by 4.3%. Meanwhile, Wu *et al.*’s experimental results also indicate a strong correlation between the size of conformational motion in 3D space and the binding strength between protein and ligand. However, the existing DL models lack dynamic structural information. Regarding this question, transforming dynamic structural information into dynamic graphs and using them as inputs for dynamic GNNs to predict that binding affinity may be a promising work [89, 90].

**Figure 7:**
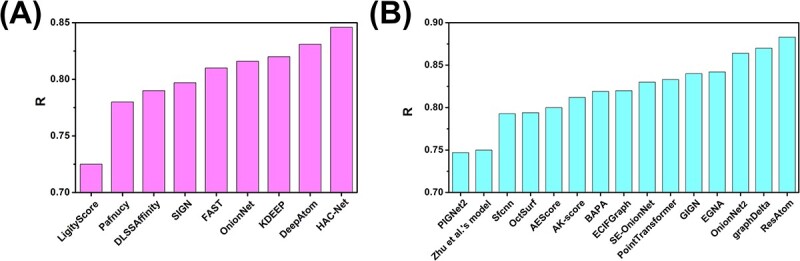
(**A**) The accuracies of nine interaction-based models on the PDBbind-2016 core set. (**B**) The accuracies of 15 interaction-based models on the CASF-2016 set.

In the model section, we classify all models into interaction-free and interaction-based models tested on the PDBbind-2016 core set or CASF-2016 set. There have been relatively few interaction-free models developed in the past 5 years. Based on the interaction-free DeepDTA model, some interaction-free models, including the DeepDTAF, DEELIG, CSConv2d, PLA-MoRe, HNN-affinity, PLANET and CAPLA, have been developed successively. The existing research results indicate that the CAPLA model has the highest accuracy (*R* = 0.843) in the PDBbind-2016 core set. The PLANET model performed the best in the CASF-2016 set with *R* = 0.824. The CAPLA is a CNN model in which the input representations of proteins, pockets and ligands are used as inputs for each of the three CNN blocks. The PLANET, a GNN model, extracts features from the 3D structure of the binding pocket and the 2D graph of the ligand molecule, respectively. A protein–ligand communication module updates these features in an across-attention manner. Compared to the DeepDTA, the CAPLA and PLANET models incorporated pocket structure information. This indicates that pocket structure information is crucial for predicting protein–ligand binding affinity. Interaction-based models have been widely studied and developed in the past 5 years. The test results indicate that HAC-Net and ResAtom System have the highest accuracy on the PDBbind-2016 core set and CASF-2016 set, respectively (Figures 7A and B) [91]. The HAC-Net, a hybrid attention-based DL model, consists of one 3D-CNN with a 3D voxel grid representation of the protein–ligand complex and two GCNs with a graph representation of the protein–ligand complex. The ResAtom, a CNN model, was built through the ResNet neural network with an attention mechanism. Both HAC-Net and ResAtom introduce the attention mechanism, which again shows that the attention mechanism can effectively improve the model’s prediction accuracy. In addition to accuracy, efficiency is also one of the critical indicators to evaluate model performance. Improving the efficiency of deep neural networks can process large-scale data efficiently and reduce the consumption of hardware resources and energy to reduce the use cost. Jin *et al*. [81] compared the efficiency of sequence-based and structure-based models, and the results showed that the sequence-based methods have more efficient computations than the structure-based methods in terms of running time. In addition, the efficiency of CAPLA was higher than that of DeepDTA and DeepDTAF. Through analysis and discussion of the accuracy and efficiency of existing models, using the CAPLA model for sequence-based protein–ligand binding affinity prediction is the optimal choice. The HAC-Net and ResAtom models should be preferred for structure-based protein–ligand binding affinity prediction.

## CONCLUSION

In the past 5 years, the prediction of protein–ligand binding affinity based on DL models has been of great interest to researchers, considering their application prospects in computer-aided drug design. Currently, a large number of DL methods have been developed to predict protein–ligand binding affinity. However, as we discussed, due to the limitations of the databases, input representation and models, the accurate prediction of protein–ligand binding affinity via DL models is still an unsolved problem. In this review, we first thoroughly analyzed and classified the databases, input representations and DL models. Then, several key challenges and future work directions were proposed to address the limitations in this rapidly expanding field.

**Key Points**

- There is an urgent need to improve the prediction accuracy of protein–ligand binding affinity for shortening cycles and reducing costs in drug development process.
- The more suitable input representation and the growing amount of experimental crystal structure and binding affinity data make the deep learning (DL) model have the potential to achieve accurate prediction of protein–ligand binding affinity in the future.
- There is a promising potential of developing new database with balance samples, dynamic structures, more protein mutants and cellular environmental information to promote the development of DL model–based methods.
- Transforming dynamic structural information into dynamic graphs and using them as inputs for dynamic graph neural networks to predict protein–ligand binding affinity may be a promising work.

## Supplementary Material

Supporting_Material_bbae081

## Author Biographies

**Huiwen Wang** is a lecturer in the School of Physics and Engineering at the Henan University of Science and Technology. Her research is focused on structural biology and deep learning.
