# Supplementary material for: Prediction of protein–ligand binding affinity via deep learning models
Source: Brief Bioinform. 2024 Mar 5;25(2):bbae081. doi: 10.1093/bib/bbae081 (PMC10939342; doi:10.1093/bib/bbae081)
Supplement: Supporting_Material_bbae081 [file supporting_material_bbae081.docx]

**Supporting Material**

**Prediction of protein-ligand binding affinity via deep learning models**

Huiwen Wang^1*^

1. School of Physics and Engineering, Henan University of Science and Technology, Luoyang 471023, China.

* Corresponding author: (huiwenwang@haust.edu.cn) H.W

**Table S1.** List of deep learning models for predicting protein-ligand binding affinity.

|  | Model | Framework | Database | | | Model Classification | Prediction accuracy | Year |
| --- | --- | --- | --- | --- | --- | --- | --- | --- |
|  |  |  | Training  set | Validation  set | Testing  set |  |  |  |
| 1 | K_DEEP_ [71] | 3D Convolutional Neural Network | PDBbind-2016 refined set (n = 3767) | N.A. | PDBbind-2016 core set (n = 290) | Interaction-based | R = 0.82,  RMSE = 1.27,  $\rho$ = 0.82 | 2018 |
|  |  |  |  |  | CSAR set that includes CSAR2012, CSAR2014, and CSAR NRC-HiQ set (n = 208) |  | Simple average:  R = 0.59,  RMSE = 1.83,  $\rho$ = 0.5 |  |
| 2 | DeepDTA [65] | 1D Convolutional Neural Network | Five-sixths of the Davis dataset (5-fold cross-validation) | | One-sixth of the Davis dataset | Interaction-free | CI = 0.878,  MSE = 0.261 | 2018 |
|  |  |  | Five-sixths of the KIBA dataset (5-fold cross-validation) | | One-sixth of the KIBA dataset |  | CI=0.863, MSE=0.194 |  |
| 3 | Pafnucy [38] | 3D Convolutional Neural Network | PDBbind-2016 general set (n = 11906) | PDBbind-2016 refined set (n = 1000) | PDBbind-2016 core set (n = 290) | Interaction-based | R = 0.78,  RMSE = 1.42,  MAE = 1.13,  SD = 1.37 | 2018 |
|  |  |  |  |  | PDBbind-2013 core set (n = 195) |  | R = 0.70,  RMSE = 1.62,  SD = 1.61 |  |
|  |  |  |  |  | Astex Diverse Set  (n = 73) |  | R = 0.57,  RMSE = 1.43,  MAE = 1.13,  SD = 1.43 |  |
| 4 | DeepBindRG [74] | ResNet Convolutional Neural Network | PDBbind-2018 general set  (n = 13500) | PDBbind-2018 general set  (n = 1000) | PDBbind-2018 general set (n = 925) | Interaction-based | R = 0.5993,  RMSE = 1.497,  MSE = 2.241,  MAE = 1.2049,  MAPE = 22.4016,  sMAPE = 9.5895 | 2019 |
|  |  |  |  |  | PDBbind-2013 core set (n = 195) |  | R = 0.6394,  RMSE = 1.817,  MSE = 3.3015,  MAE = 1.4829,  MAPE = 28.8105,  sMAPE = 11.9433 |  |
|  |  |  |  |  | CSAR HiQ-NRC set (n = 343) |  | R = 0.6585,  RMSE = 1.7239,  MSE = 2.9719,  MAE = 1.3607,  MAPE = 63.0363,  sMAPE = 11.1805 |  |
|  |  |  |  |  | Astex Diverse Set (n = 74) |  | R = 0.4657,  RMSE = 1.6209,  MSE = 2.6274,  MAE = 1.3355,  MAPE = 20.7896,  sMAPE = 9.9863 |  |
| 5 | OnionNet [76] | 2D Convolutional Neural Network | PDBbind-2016 general set  (n = 11906) | PDBbind-2016 refined set (n = 1000) | PDBbind-2016 core set (n = 290) | Interaction-based | R = 0.816,  RMSE = 1.278,  MAE = 0.984,  SD = 1.257 | 2019 |
|  |  |  |  |  | PDBbind-2013 core set (n = 108) |  | R = 0.782,  RMSE = 1.503,  MAE = 1.208,  SD = 1.445 |  |
| 6 | Zhu *et al*.’s model [92] | Fully Connected Neural Network | PDBbind-2018 refined set  (n = 2675) | PDBbind-2018 refined set  (n = 892) | PDBbind-2018 refined set (n = 891) | Interaction-based | R = 0.66,  RMSE = 1.61,  $\rho$ = 0.67 | 2020 |
|  |  |  |  |  | CASF-2016 (n = 285) |  | R = 0.75,  RMSE = 1.44,  $\rho$ = 0.75 |  |
| 7 | DeepAtom [72] | 3D Convolutional Neural Network | PDBbind-2016 general set (n = 9383) | PDBbind-2016 refined set (n = 1000) | PDBbind-2016 core set (n = 290) | Interaction-based | R = 0.831,  RMSE = 1.232,  MAE = 0.904,  SD = 1.222 | 2020 |
|  |  |  |  |  | Astex Diverse Set (n = 64) |  | R = 0.768,  RMSE = 1.027,  MAE = 0.714,  SD = 1.003 |  |
| 8 | AK-score [79] | an ensemble of 3D Convolutional Neural Networks | PDBbind-2016 refined set (n = 3772) | N.A. | CASF-2016 (n = 285) | Interaction-based | R = 0.812,  $\rho$= 0.670 | 2020 |
| 9 | graphDelta [73] | Graph Convolutional Neural Network | PDBbind-2018 general set (n = 8766, fivefold cross-validation) | | CASF-2016 (n = 285) | Interaction-based | R = 0.87,  RMSE = 1.05 | 2020 |
|  |  |  |  |  | CSAR NRC-HiQ set 1 (n = 53) |  | R = 0.74,  RMSE = 1.59 |  |
|  |  |  |  |  | CSAR NRC-HiQ set 2 (n = 49) |  | R = 0.71,  RMSE = 1.53 |  |
|  |  |  |  |  | CSAR2012 |  | R = 0.48,  RMSE = 1.14 |  |
|  |  |  |  |  | CSAR2014 |  | R = 0.74,  RMSE = 1.22 |  |
| 10 | FAST [80] | A fusion model consists of a 3D Convolutional Neural Network and a Spatial Graph Convolutional Neural Network | PDBbind-2016 general set (n = 13283) | | PDBbind-2016 core set (n = 290) | Interaction-based | R= 0.810,  r^2^ = 0.638,  RMSE = 1.308,  MAE = 1.019,  $\rho$ = 0.807 | 2021 |
| 11 | DeepDTAF [69] | 1D Convolutional Neural Network | PDBbind-2016 general set (n = 11906) | PDBbind-2016 refined set (n = 1000) | PDBbind-2016 core set (n = 290) | Interaction-free | R = 0.789,  RMSE = 1.355,  MAE = 1.073,  SD = 1.337,  CI = 0.799 | 2021 |
| 12 | SIGN [81] | a structure-aware interactive graph neural network | The protein-ligand complexes in PDBbind-2016 refined set (n = 3767) were randomly divided into training and validation sets with a ratio of 9:1. | | PDBbind-2016 core set (n = 290) | Interaction-based | R = 0.797,  RMSE = 1.316,  MAE = 1.027,  SD = 1.312 | 2021 |
|  |  |  |  |  | CSAR HiQ set (n = 343) |  | R = 0.754,  RMSE = 1.735,  MAE = 1.327,  SD = 1.709 |  |
| 13 | LigityScore [93] | Convolutional Neural Network | PDBbind-2016 general set (n = 11906) | PDBbind-2016 refined set (n = 1000) | PDBbind-2013 core set (n = 108) | Interaction-based | R = 0.713,  RMSE = 1.676,  MAE = 1.335,  SD = 1.583 | 2021 |
|  |  |  |  |  | PDBbind-2016 core set (n = 290) |  | R = 0.725,  RMSE = 1.509,  MAE = 1.224,  SD = 1.497 |  |
| 14 | BAPA [94] | Three kinds of neural network layers (convolutional, attention, and dense) | PDBbind-2016 refined set (n = 3689) | PDBbind-2018 refined set  (n = 677) | CASF-2016 (n = 285) | Interaction-based | R = 0.819,  RMSE = 1.308,  MAE = 1.021,  $\rho$ = 0.819,  SD = 1.247 | 2021 |
|  |  |  |  |  | CASF-2013 (n = 195) |  | R = 0.771,  RMSE = 1.457,  MAE = 1.170,  $\rho$ = 0.774,  SD = 1.433 |  |
|  |  |  |  |  | CSAR NRC-HiQ set 1 (n = 50) |  | R = 0.826,  RMSE = 1.453,  MAE = 1.060,  $\rho$ = 0.827,  SD = 1.329 |  |
|  |  |  |  |  | CSAR NRC-HiQ set 2 (n = 44) |  | R = 0.775,  RMSE = 1.294,  MAE = 0.982,  $\rho$ = 0.782,  SD = 1.294 |  |
| 15 | DEELIG [70] | Convolutional Neural Network | A database (n = 4041) created by the author is divided into training, validation, and test sets in a ratio of 80:10:10. | | Test set | Interaction-free | RMSE = 3.07,  MAE = 2.27,  SD = 2.06,  PCC = 0.794 | 2021 |
|  |  |  |  |  | CASF-2016 (n = 285) |  | R = 0.889 |  |
|  |  |  |  |  | CASF-2013 (n = 195) |  | R = 0.894 |  |
| 16 | ResAtom System [95] | The ResAtom System consists of a convolution layer, an attention block, sixteen basic residual blocks, and a fully connected layer. | PDBbind-2016 general set (n = 15038). The 15038 complexes were divided into training and validation sets in a ratio of 80:20. | | CASF-2016 (n = 285) | Interaction-based | R = 0.883 | 2021 |
|  |  |  |  |  | CSAR HiQ set (n = 75) |  | R = 0.52,  RMSE = 2.19,  MAE = 1.72 |  |
| 17 | SE-OnionNet [96] | SE-OnionNet consists of OnionNet and squeeze-and-excitation (SE) blocks. | PDBbind-2018 general set (n = 11663) | PDBbind-2018 refined set (n = 4000) | CASF-2018  (n = 463) | Interaction-based | R = 0.853,  RMSE = 1.592,  MAE = 0.912,  SD = 1.253 | 2021 |
|  |  |  |  |  | CASF-2016 (n = 285) |  | R = 0.83,  SD = 1.20 |  |
|  |  |  |  |  | CASF-2013  (n = 195) |  | R = 0.812,  RMSE = 1.692,  MAE = 1.323,  SD = 1.423 |  |
| 18 | OnionNet-2 [97] | 2D Convolutional Neural Network | PDBbind-2019 general set | PDBbind-2019 refined set (n = 1000) | CASF-2016 (n = 285) | Interaction-based | R = 0.864,  RMSE = 1.164 | 2021 |
|  |  |  |  |  | CASF-2013 (n = 195) |  | R = 0.821,  RMSE = 1.357 |  |
|  |  |  |  |  | CSAR NRC-HiQ set 1 (n = 55) |  | R = 0.89,  RMSE = 1.50 |  |
|  |  |  |  |  | CSAR NRC-HiQ set 2 (n = 49) |  | R = 0.87,  RMSE = 1.21 |  |
| 19 | OctSurf [98] | 3D Convolutional Neural Networks | PDBbind-2018 general | PDBbind-2018 refined set (n = 600) | CASF-2016 (n = 285) | Interaction-based | r^2^ = 0.63,  RMSE = 1.45,  MAE = 1.16,  $\rho$ = 0.79,  $\tau$ = 0.59 | 2021 |
| 20 | AEScore [99] | Feed-forward neural network | PDBbind-2016 refined set (n = 3464) | PDBbind-2016 refined set (n = 385) | CASF-2016 (n = 285) | Interaction-based | R= 0.80,  RMSE = 1.30,  $\rho$ = 0.80 | 2021 |
|  |  |  |  |  | CASF-2013 (n = 195) |  | R= 0.76,  RMSE = 1.46,  $\rho$ = 0.76 |  |
| 21 | CSConv2d [100] | A 2D Structural Convolutional Neural Network | ChEMBLv23 dataset (n = 905828, The positive and negative sample ratio is close to 1:1, which is 475238 and 430590, respectively) | | ChEMBL Bioactivity Benchmark Set | Interaction-free | Matthews correlation coefficient (MCC) = 0.57 | 2021 |
| 22 | DLSSAffinity [83] | Convolutional Neural Network | PDBbind-2016 general set (n = 11906) | PDBbind-2016 refined set (n = 1000) | PDBbind-2016 core set (n = 290) | Interaction-based | R = 0.79,  RMSE = 1.40,  SD = 1.35 | 2022 |
|  |  |  |  |  | CASF-2013 (n = 195) |  | RMSE = 1.60 |  |
| 23 | PLA-MoRe [66] | A  Fully Connected Network | Five-sixths of the Davis dataset (n = 27404, fivefold cross-validation) | | One-sixth of the Davis dataset (n = 27404) | Interaction-free | RMSE = 0.483,  MSE = 0.234,  CI = 0.886 | 2022 |
|  |  |  | Five-sixths of the KIBA dataset (n = 107946, fivefold cross-validation) | | One-sixth of the KIBA dataset (n = 107946) |  | RMSE = 0.396,  MSE = 0.157,  CI = 0.874 |  |
|  |  |  | Five-sixths of the PDBbind-2016 dataset (n = 4591, fivefold cross validation) | | One-sixth of the PDBbind-2016 dataset (n = 4591) |  | RMSE = 1.230,  MSE = 1.515,  CI = 0.780 |  |
| 24 | HNN-denovo [67] | A Hybrid Neural Network Framework, including the 3D convolutional neural network and the fast-forward neural network | PDBbind-2019 refined set (n = 4357) | | PDBbind-2019 refined set (n = 300) | Interaction-based | R= 0.86,  RMSE = 1.11 | 2022 |
|  |  |  | PDBbind-2019 refined set (n = 4357) | | PDBbind-2019 refined set (n = 797) |  | R= 0.84,  RMSE = 0.96 |  |
| 25 | HNN-affinity [66] | A Hybrid Neural Network Framework including the 3D convolutional neural network and the fast forward neural network | PDBbind-2019 refined set (n = 3860) | | PDBbind-2019 refined set (n = 300) | Interaction-free | R= 0.83,  RMSE = 1.04 | 2022 |
|  |  |  | PDBbind-2019 refined set (n = 3860) | | PDBbind-2019 refined set (n = 797) |  | R= 0.82,  RMSE = 1.06 |  |
| 26 | Sfcnn [101] | 3D Convolutional Neural Network | PDBbind-2016 refned set (n = 4100) | PDBbind-2016 refned set (n = 486) | CASF-2016 (n = 285) | Interaction-based | R = 0.7928,  RMSE = 1.3263,  MAE = 1.0277,  SD = 1.3253 | 2022 |
|  |  |  |  |  | CASF-2013 (n = 107) |  | R = 0.7946,  RMSE = 1.4518,  MAE = 1.1139,  SD = 1.4165 |  |
|  |  |  |  |  | CSAR HiQ-NRC set (n = 343) |  | R = 0.824,  RMSE = 1.277,  MAE = 0.8375 |  |
|  |  |  |  |  | CSAR HiQ-NRC set* (n = 149) |  | R = 0.6758,  RMSE = 1.8079,  MAE = 1.3680 |  |
|  |  |  |  |  | Astex Diverse set (n = 74) |  | R = 0.6474,  RMSE = 1.3627,  MAE = 1.0518 |  |
| 27 | DeepBindBC [102] | Convolutional Neural Network | Positive data: PDBbind-2018 general set (n = 13500)  Negative data (n = 13500) | Positive data: PDBbind-2018 general set (n = 1000)  Negative data (n = 1000) | Positive data: PDBbind-2018 general set (n = 925)  Negative data (n = 925) | Interaction-based | AUC = 0.93,  Accuracy = 0.85,  TPR = 0.86,  Precision = 0.84 | 2022 |
|  |  |  |  |  | Positive data: CASF-2013 (n = 195)  Negative data (n = 195) |  | AUC = 0.92,  Accuracy = 0.82,  TPR = 0.90,  Precision = 0.78 |  |
|  |  |  |  |  | Positive data: CSAR HiQ-NRC set (n = 343)  Negative data (n = 343) |  | AUC = 0.72,  Accuracy = 0.71,  TPR = 0.54,  Precision = 0.82 |  |
|  |  |  |  |  | Positive data: Astex Diverse set (n = 74)  Negative data (n = 74) |  | AUC = 0.87,  Accuracy = 0.78,  TPR = 0.81,  Precision = 0.77 |  |
| 28 | PIGNet [77] | Fully Connected Network | PDBbind-2019 refined set (n = 4514) | N.A. | PDBbind-2016 core set (n = 283) | Interaction-based | R= 0.761,  $\rho$ = 0.682 | 2022 |
|  |  |  |  |  | CSAR HiQ-NRC set 1 (n = 37) |  | R= 0.768 |  |
|  |  |  |  |  | CSAR HiQ-NRC set 2 (n = 22) |  | R= 0.800 |  |
| 29 | PointTransformer [103] | A point cloud-based neural network structures | PDBbind-2016 refined set (n = 3772) | | CASF-2016 (n = 285) | Interaction-based | R= 0.753,  RMSE = 1.58,  MAE = 1.29,  $\rho$ = 0.751 | 2022 |
|  |  |  | extended set (n = 11327).  The 11327 complexes were divided into training and validation sets in ratio 90:10. | | CASF-2016 (n = 285) |  | R= 0.833,  RMSE = 1.26,  MAE = 0.99,  $\rho$ = 0.825 |  |
| 30 | PIGNet2 [104] | Fully Connected Network | PDBbind-2019 refined set (n = 5046) | N.A. | CASF-2016 (n = 285) | Interaction-based | R= 0.747,  $\rho$ = 0.651 | 2023 |
| 31 | PLANET [68] | Graph Neural Network | PDBbind-2020 general set (n = 15616) | PDBbind-2020 general set (n = 3401) | CASF-2016 (n = 285) | Interaction-free | R = 0.824,  RMSE = 1.247,  MAE = 0.965 | 2023 |
| 32 | ECIFGraph [78] | Graph Neural Network | PDBbind-2016 refined set (n = 3772) | | CASF-2016 (n = 285) | Interaction-based | R = 0.820,  RMSE = 1.270,  $\rho$ = 0.675 | 2023 |
| 33 | GraphscoreDTA [86] | Graph Neural Network | PDBbind-2019 general set (n = 13851, The ratios of clusters in the training, validation and test sets were 7:1:2.) | | CASF-2016 (based on compound similarity clustering, n = 279) | Interaction-based | R = 0.831,  RMSE = 1.249,  MAE = 0.981,  SD = 1.216,  CI = 0.819 | 2023 |
|  |  |  |  |  | CASF-2016 (based on protein similarity clustering, n = 279) |  | R = 0.810,  RMSE = 1.349,  MAE = 1.053,  SD = 1.281,  CI = 0.811 |  |
|  |  |  |  |  | CASF-2013 (based on compound similarity clustering, n = 182) |  | R = 0.831,  RMSE = 1.249,  MAE = 0.981,  SD = 1.216,  CI = 0.819 |  |
|  |  |  |  |  | CASF-2013 (based on protein similarity clustering, n = 182) |  | R = 0.757,  RMSE = 1.486,  MAE = 1.179,  SD = 1.480,  CI = 0.782 |  |
| 34 | CAPLA [79] | Convolutional Neural Network | PDBbind-2016 general set (n = 11906) | PDBbind-2016 refined set (n = 1000) | CASF-2016 core set  (n = 290) | Interaction-free | R = 0.843,  RMSE = 1.200,  MAE = 0.966,  SD = 1.170,  CI = 0.820 | 2023 |
|  |  |  |  |  | CASF-2013 (n = 195) |  | R = 0.770,  RMSE = 1.466,  MAE = 1.155,  SD = 1.436,  CI = 0.780 |  |
|  |  |  |  |  | CSAR HiQ set 1 (n = 51) |  | R = 0.686,  RMSE = 1.848,  MAE = 1.550,  SD = 1.701,  CI = 0.727 |  |
|  |  |  |  |  | CSAR HiQ set 2 (n = 36) |  | R = 0.704,  RMSE = 1.454,  MAE = 1.160,  SD = 1.420,  CI = 0.760 |  |
| 35 | EGNA [75] | Graph Neural Network | PDBbind-2016 general set | | CASF-2016 (n = 285) | Interaction-based | R = 0.842,  RMSE = 1.258,  MAE = 0.980 | 2023 |
|  |  |  |  |  | CSAR HiQ set (n = 49) |  | R = 0.750,  RMSE = 1.536,  MAE = 1.190 |  |
| 36 | GIGN [87] | Graph Neural Network | PDBbind-2016 general set (n = 11904) | PDBbind-2016 general set (n = 1000) | PDBbind-2019 holdout set (n = 4366) | Interaction-based | R = 0.641,  RMSE = 1.393 | 2023 |
|  |  |  |  |  | CASF-2016 (n = 285) |  | R = 0.840,  RMSE = 1.190 |  |
|  |  |  |  |  | PDBbind-2013 core set (n = 107) |  | R = 0.821,  RMSE = 1.380 |  |
|  |  |  |  |  | CSAR HiQ set (n = 47) |  | R = 0.760,  RMSE = 1.538 |  |
| 37 | HAC-Net [105] | A Hybrid Attention-Based Convolutional Neural Network | PDBbind-2020 general set (n = 18108) | PDBbind-2020 general set (n = 300) | PDBbind-2016 core set (n = 290) | Interaction-based | R= 0.846,  r^2^ = 0.692,  RMSE = 1.205,  MAE = 0.971,  $\rho$ = 0.843 | 2023 |

**Table S2.** The numbers of all complexes with binding affinity in general set, refined set, and core set for each version of PDBbind database.

| Version | All Complexes with Binding Affinity | | |
| --- | --- | --- | --- |
|  | General Set | Refined Set | Core Set |
| 2002 | 1446 | 800 | N.A. |
| 2003 | 1763 | 900 | N.A. |
| 2004 | 2276 | 1091 | 231 |
| 2005 | 2756 | 1296 | 288 |
| 2006 | 2632 | 1122 | 234 |
| 2007 | 3124 | 1300 | 210 |
| 2008 | 4300 | 1401 | 210 |
| 2009 | 5678 | 1741 | 219 |
| 2010 | 6772 | 2061 | 231 |
| 2011 | 7986 | 2455 | 216 |
| 2012 | 9308 | 2897 | 201 |
| 2013 | 10776 | 2959 | 195 |
| 2014 | 12995 | 3446 | 195 |
| 2015 | 14620 | 3706 | 195 |
| 2016 | 16179 | 4057 | 290 |
| 2017 | 17900 | 4154 | 285 |
| 2018 | 19588 | 4463 | 285 |
| 2019 | 21382 | 4852 | 285 |
| 2020 | 23496 | 5316 | 285 |
